# Bibliographical

**Published:** 1880-09

**Authors:** 


### BIBLIOGRAPHICAL.

The third edition of Richardson's Mechanical Dentist is now in press, and will be issued within a brief period. Those familiar with the former editions of this work, and especially those who know Dr. Richardson, know well what to expect. The work will certainly be fully abreast with the attainments of the time.

This work has heretofore deservedly stood at the head, as a text book on this subject, and it is not extravagant to say that it will still stand there. We hope soon to see it.

